# Supplementary material for: Repeated introduction of micropollutants enhances microbial succession despite stable degradation patterns
Source: ISME Commun. 2022 Jun 7;2:48. doi: 10.1038/s43705-022-00129-0 (PMC9723708; doi:10.1038/s43705-022-00129-0)

Supplemental information for

Repeated introduction of micropollutants enhances microbial succession despite stable degradation patterns

Running title: Ecological feedback between microbe-micropollutants

Dandan Izabel-Shen[1#], Shuang Li[2,3,4#], Tingwei Luo[5], Jianjun Wang[6], Yan Li[2,3], Qian Sun[2,3], Chang-Ping Yu[2,7], Anyi Hu[2,3*]

1. Department of Ecology, Environment and Plant Sciences, Stockholm University, 106 91, Stockholm, Sweden
2. CAS Key Laboratory of Urban Pollutant Conversion, Institute of Urban Environment, Chinese Academy of Sciences, Xiamen 361021, China
3. University of Chinese Academy of Sciences, Beijing 100049, China
4. Department of Environmental Microbiology, UFZ, Helmholtz Centre for Environmental Research, Leipzig, Germany
5. Institute of Marine Microbes and Ecospheres, State Key Laboratory of Marine Environmental Science, Xiamen University, Xiamen, China
6. State Key Laboratory of Lake Science and Environment, Nanjing Institute of Geography and Limnology, Chinese Academy of Sciences, Nanjing, 210008, China
7. Graduate Institute of Environmental Engineering, National Taiwan University, Taipei 106, Taiwan

#The authors contributed equally to this work.

*Correspondence to:

Dr. Anyi Hu, ayhu@iue.ac.cn

CAS Key Laboratory of Urban Pollutant Conversion, Institute of Urban Environment, Chinese Academy of Sciences, Xiamen 361021, China, Telephone ＆ Fax: 86-592-6190582.

Contents:

Supplemental text	
Part1) Additional experiments to test micropollutant degradation	Page 3
Part2) Calculation of the weighted mean rRNA gene copy number	Page 4
Part3) Analysis of line regression model	Page 5
Supplemental Tables	
Table S1	Page 6
Table S2	Page 7
Table S3	Pages 8-14
Table S4	Pages 15-18
Supplemental Figures	
Figure S1	Page 19
Figure S2	Page 20
Figure S3	Page 21
Figure S4	Page 22
Figure S5	Page 23
Figure S6	Page 24
Figure S7	Page 25
Figure S8	Page 26
Figure S9	Page 27

Supplemental methods

Part 1) Additional experiments to test micropollutant degradation

An additional experiment was conducted to test the removal rates of micropollutants in the absence of microorganisms. Reservoir water was filtered through 0.22-μm filters and then amended with a mixture consisting of 1 μg BPA/L, 0.1 μg BPS/L, 1 μg TCS/L, and 0.1 μg TCC/L as a blank control. A second additional experiment was conducted to assess whether the ability of prokaryotic assemblages to degrade BPA and TCS was enhanced by the evolving community succession following micropollutant addition. Thus, 40 ml of the initial inoculum and 40 ml of the bacterial assemblage at the end of the experiment (after incubation of the B7 microcosms) were each amended with 160 ml of BPA, 160 ml of TCS, or a mixture thereof, with each micropollutant present at a final concentration of 1 mg/L. The removal rates of BPA and TCS in these treatments after 7 days were measured and the rates in the initial inocula vs. those in the B7 microcosms were compared. The incubation conditions were the same for each treatment and the experiment was conducted in triplicate.

The first experiment showed that the removal proportion of the four micropollutants in the prokaryote-free controls was stable during the 7-day incubation (Supplemental Fig. S2), and therefore the chemical stability and negligible transformation of the micropollutants in the absence of microorganisms. The second experiment showed that the concentration of BPA declined later in the initial community without pre-exposure than in the community with a 35-day history of exposure (Supplemental Fig. S3). In the case of TCS, the concentration did not change over time in the initial community, while in the pre-exposed community it decreased sharply at day 4 of the incubation. These results demonstrated that pre-exposure to micropollutants impacted the ability of the bacterial community to transform micropollutants during a later exposure and that this ability differed between communities exposed to BPA and TCS.

Part 2) Calculation of the weighted mean rRNA gene copy number

The rrnDB database catalogues the 16S rRNA gene copy number of an organism with its NCBI or RDP taxonomy [1]. The 16S rRNA gene copy number of an OTU is estimated to be the number of its assigned genus. If the 16S rRNA gene copy number of the corresponding genus is not provided in the rrnDB database, then number of the higher phylogenetic level (i.e., family/order/class/phylum) is used. The abundance-weighed average gene copy number N_{rrn} of each community is calculated using the relative abundance P of each OTU in the community, the taxonomic assignment of each OTU, and the estimated 16S rRNA gene copy number n of each OTU based on rrnDB database v5.5 [2]:

$$N_{rrn} = \sum_{i=1}^{x} (n_i \times P_i)$$

where n_i is the estimated 16S rRNA gene copy number of OTU i, P_i is the relative abundance of OTU i, and x is the total number of OTU in the microbial community.

Part 3) Analysis of line regression model

A linear regression model was used to evaluate the rate of community turnover over time:

$$D = vT$$

where D is the Bray-Curtis dissimilarity between the micropollutant treated vs. untreated communities, T is the incubation time, and the slope v is the turnover rate. The time-decay relationship (TDR) of the PTC of each treatment group and control was evaluated using a log-transformed power law model [3,4]:

$$\log(S) = c + w \log(T)$$

where S is the pairwise Bray-Curtis similarity in community composition across time interval T, and the slope w is the temporal turnover rate of the community within each treatment group or control. The significance of the values of slopes v and w was evaluated by 1,000 permutations, using the R package lmPerm [5]. Significant inter-treatment differences in those values were tested by 1,000 bootstraps, followed by a pairwise t-test with Bonferroni correction [6,7].

References

1. Stoddard SF, Smith BJ, Hein R, Roller BRK and Schmidt TM. rrnDB: improved tools for interpreting rRNA gene abundance in bacteria and archaea and a new foundation for future development. *Nucleic Acids Research*. 2015; 43:593–598.
2. Klappenbach JA, Saxman PR, Cole JR, Schmidt TM. Rrndb: The ribosomal RNA operon copy number database. *Nucleic Acids Res.* 2001; 29:181–184.
3. Guo X, Feng J, Shi Z, Zhou X, Yuan M, Tao X, et al. Climate warming leads to divergent succession of grassland microbial communities. *Nat Clim Change*. 2018; 8:813–818.
4. Qian J, Ding Q, Guo A, Zhang D, Wang K. Alteration in successional trajectories of bacterioplankton communities in response to co-exposure of cadmium and phenanthrene in coastal water microcosms. *Environ Pollut*. 2017; 221:480–490.
5. Wheeler B, Torchiano M. lmPerm: Permutation tests for linear models. R package version 2010, 1, (v1.2). Available from: https://cran.r-project.org/package=vegan
6. Zhou J, Kang S, Schadt CW, Garten Jr CT. Spatial scaling of functional gene diversity across various microbial taxa. *Proc Natl Acad Sci U S A*. 2008; 105:7768–7773
7. Liang Y, Jiang Y, Wang F, Wen C, Deng Y, Xue K, et al. Long-term soil transplant simulating climate change with latitude significantly alters microbial temporal turnover. *ISME J*. 2015; 9: 2561–2572.

Tables

Table S1. Explanatory values of the environmental variables to differences in the communities.

Explanatory values

	NMDS1	*NMDS2*	R^2	*P value*
BPA	0.22	0.98	0.05	0.06*
BPS	0.22	-0.97	0.02	0.59
TCC	-0.1	-0.99	0.02	0.29
TCS	0.1	1	0.24	0.001**

Significance codes: ‘**’ 0.01, ‘*’ 0.1.

Number of permutations: 999

Table S2. The results of Permutational Multivariate Analysis of Variance (PERMANOVA) testing the quantitative effects of treatment (i.e., the controls and the type of micropollutant), incubation time (corresponding to microcosm batches), and their interactions on the bacterial community structures across all samples and between treatment pair. R^2 values represent the proportion of variance explaining community variation. Bold R^2 and P values represent significance of the variance at $P < 0.05$. Treatment IDs are: 'Con' microcosms containing no micropollutant additions and serve as the controls, 'BPA' microcosms containing bisphenol A, 'TCS' microcosms containing triclosan, 'MI' microcosms containing a mixture of bisphenol A and triclosan, 'MII' microcosms containing a mixture of bisphenol A, triclosan, bisphenol-S and triclocarban.

PERMANOVA			pairwise PERMANOVA		
Factor	R^2	*P*-value	Pairs	R^2	*P*-value
Incubation time	**0.149**	**0.001**	Con. vs BPA	0.042	0.6
Treatment	**0.11**	**0.001**	Con. vs TCS	**0.108**	**0.01**
Incubation time *Treatment	**0.055**	**0.001**	Con. vs MI	**0.1**	**0.01**
			Con. vs MII	**0.095**	**0.01**
			BPA vs TCS	**0.077**	**0.02**
			BPA vs MI	0.067	0.06
			BPA vs MII	**0.072**	**0.01**
			TCS vs MI	0.038	1
			TCS vs MII	0.068	0.12
			MI vs MII	0.042	0.79

Table S3 The ecological strategies of microbial taxa that responded to repeated introduction of micropollutants exhibited distinct response trajectories. Here, the list of OTUs assigned to ecological groups: "opportunistic", "sensitive" and "tolerant" taxa in each micropollutant treatment.

Ecological category	Treatment	OTUID	Taxonomy (Phylum/class)	log2(P1/P2)	*P.adj*	log2(P2/P3)	*P.adj*
opportunistic	BPA	OTU_129	Bacteroidetes	-8.06	0.000	4.58	0.026
opportunistic	TCS	OTU_376	Alphaproteobacteria	-4.67	0.001	3.41	0.008
opportunistic	MI	OTU_53	Planctomycetes	-5.33	0.000	3.70	0.013
opportunistic	MI	OTU_167	Proteobacteria	-8.91	0.003	6.64	0.027
opportunistic	MI	OTU_126	Deltaproteobacteria	-5.09	0.019	4.44	0.044
sensitive	BPA	OTU_60	Actinobacteria	26.79	0.000	11.67	0.000
sensitive	BPA	OTU_169	Actinobacteria	30.00	0.000	24.26	0.000
sensitive	BPA	OTU_3	Planctomycetes	8.65	0.000	4.47	0.001
sensitive	BPA	OTU_27	Actinobacteria	12.65	0.000	12.23	0.000
sensitive	BPA	OTU_47	Actinobacteria	11.77	0.000	11.68	0.000
sensitive	BPA	OTU_534	Gammaproteobacteria	22.90	0.000	23.05	0.000
sensitive	BPA	OTU_19	Planctomycetes	10.49	0.000	5.72	0.003
sensitive	BPA	OTU_185	Bacteroidetes	5.64	0.000	5.26	0.000
sensitive	BPA	OTU_107	Gammaproteobacteria	2.71	0.000	2.29	0.000
sensitive	BPA	OTU_81	Actinobacteria	10.78	0.000	9.88	0.000
sensitive	BPA	OTU_202	Alphaproteobacteria	5.98	0.000	4.91	0.000
sensitive	BPA	OTU_52	Planctomycetes	10.40	0.000	7.04	0.001
sensitive	BPA	OTU_76	Gammaproteobacteria	4.67	0.000	4.30	0.000
sensitive	BPA	OTU_117	Bacteroidetes	7.65	0.000	5.22	0.002
sensitive	BPA	OTU_65	Actinobacteria	11.17	0.000	11.07	0.000
sensitive	BPA	OTU_222	Bacteroidetes	9.12	0.000	8.54	0.000
sensitive	BPA	OTU_136	Planctomycetes	4.40	0.000	6.34	0.000
sensitive	BPA	OTU_187	Alphaproteobacteria	5.00	0.000	2.95	0.013
sensitive	BPA	OTU_131	Alphaproteobacteria	4.90	0.000	3.39	0.005
sensitive	BPA	OTU_266	Bacteroidetes	6.16	0.000	7.32	0.000
sensitive	BPA	OTU_165	Bacteroidetes	9.10	0.000	5.60	0.012
sensitive	BPA	OTU_9	Alphaproteobacteria	2.80	0.001	1.73	0.025
sensitive	BPA	OTU_98	Verrucomicrobia	3.77	0.002	4.56	0.000
sensitive	BPA	OTU_366	Bacteroidetes	8.02	0.002	7.45	0.001
sensitive	BPA	OTU_397	Alphaproteobacteria	8.57	0.003	6.97	0.005
sensitive	BPA	OTU_104	Actinobacteria	9.34	0.004	8.77	0.001

Ecological category	Treatment	OTUID	Taxonomy (Phylum/class)	log2(P1/P2)	*P.adj*	log2(P2/P3)	*P.adj*
sensitive	BPA	OTU_74	Actinobacteria	9.71	0.005	8.81	0.003
sensitive	BPA	OTU_113	Planctomycetes	3.88	0.005	6.13	0.000
sensitive	BPA	OTU_135	Alphaproteobacteria	8.95	0.005	8.38	0.002
sensitive	BPA	OTU_245	Bacteroidetes	9.26	0.005	7.15	0.013
sensitive	BPA	OTU_154	Verrucomicrobia	5.57	0.006	4.58	0.009
sensitive	BPA	OTU_249	Alphaproteobacteria	3.45	0.006	2.63	0.017
sensitive	BPA	OTU_203	Verrucomicrobia	7.53	0.006	6.63	0.005
sensitive	BPA	OTU_164	Gammaproteobacteria	6.68	0.007	8.38	0.000
sensitive	BPA	OTU_413	Planctomycetes	5.20	0.010	6.03	0.000
sensitive	BPA	OTU_137	Actinobacteria	9.44	0.010	8.87	0.005
sensitive	BPA	OTU_178	Verrucomicrobia	9.18	0.011	8.61	0.005
sensitive	BPA	OTU_268	Gammaproteobacteria	3.59	0.011	4.22	0.001
sensitive	BPA	OTU_72	Gammaproteobacteria	3.38	0.011	3.48	0.003
sensitive	BPA	OTU_323	Gammaproteobacteria	3.53	0.013	3.15	0.011
sensitive	BPA	OTU_462	Bacteroidetes	7.97	0.014	7.39	0.008
sensitive	BPA	OTU_90	Actinobacteria	8.09	0.014	7.99	0.005
sensitive	BPA	OTU_160	Actinobacteria	9.16	0.014	8.59	0.008
sensitive	BPA	OTU_446	Actinobacteria	6.23	0.015	5.65	0.010
sensitive	BPA	OTU_357	Bacteroidetes	4.74	0.020	6.07	0.001
sensitive	BPA	OTU_335	Bacteroidetes	8.78	0.020	8.21	0.012
sensitive	BPA	OTU_170	Bacteroidetes	5.27	0.024	7.43	0.000
sensitive	BPA	OTU_256	Bacteroidetes	5.25	0.025	6.07	0.003
sensitive	BPA	OTU_278	Verrucomicrobia	6.70	0.028	6.12	0.019
sensitive	BPA	OTU_379	Actinobacteria	6.93	0.029	6.36	0.020
sensitive	BPA	OTU_195	Alphaproteobacteria	3.85	0.030	4.53	0.004
sensitive	BPA	OTU_274	Verrucomicrobia	6.44	0.030	5.86	0.022
sensitive	BPA	OTU_166	Gammaproteobacteria	3.53	0.030	3.41	0.017
sensitive	BPA	OTU_285	Cyanobacteria	5.71	0.034	4.82	0.039
sensitive	BPA	OTU_149	Planctomycetes	2.58	0.036	4.99	0.000
sensitive	BPA	OTU_158	Actinobacteria	7.93	0.036	7.35	0.025
sensitive	BPA	OTU_234	Bacteroidetes	2.94	0.040	3.50	0.005
sensitive	BPA	OTU_313	Bacteroidetes	4.60	0.040	6.97	0.001
sensitive	BPA	OTU_439	Verrucomicrobia	6.10	0.040	5.52	0.033
sensitive	BPA	OTU_227	Verrucomicrobia	7.58	0.040	7.01	0.030
sensitive	BPA	OTU_388	Gammaproteobacteria	4.46	0.042	6.03	0.002
sensitive	BPA	OTU_308	Actinobacteria	7.58	0.044	7.00	0.035
sensitive	BPA	OTU_470	Gammaproteobacteria	4.10	0.047	4.96	0.005
sensitive	BPA	OTU_493	Planctomycetes	4.19	0.049	4.26	0.020
sensitive	TCS	OTU_74	Actinobacteria	25.10	0.000	25.31	0.000
sensitive	TCS	OTU_110	Actinobacteria	25.81	0.000	26.19	0.000
sensitive	TCS	OTU_158	Actinobacteria	23.37	0.000	23.92	0.000

Ecological category	Treatment	OTUID	Taxonomy (Phylum/class)	log2(P1/P2)	*P.adj*	log2(P2/P3)	*P.adj*
sensitive	TCS	OTU_201	Verrucomicrobia	23.15	0.000	23.85	0.000
sensitive	TCS	OTU_27	Actinobacteria	11.04	0.000	11.47	0.000
sensitive	TCS	OTU_47	Actinobacteria	9.30	0.000	10.73	0.000
sensitive	TCS	OTU_185	Bacteroidetes	4.80	0.000	7.53	0.000
sensitive	TCS	OTU_117	Bacteroidetes	4.31	0.000	7.20	0.000
sensitive	TCS	OTU_104	Actinobacteria	8.59	0.001	9.57	0.000
sensitive	TCS	OTU_165	Bacteroidetes	9.55	0.001	9.56	0.000
sensitive	TCS	OTU_72	Gammaproteobacteria	8.60	0.001	8.76	0.000
sensitive	TCS	OTU_131	Alphaproteobacteria	4.08	0.002	4.70	0.000
sensitive	TCS	OTU_332	Bacteroidetes	6.77	0.002	7.26	0.000
sensitive	TCS	OTU_187	Alphaproteobacteria	3.77	0.002	3.41	0.001
sensitive	TCS	OTU_81	Actinobacteria	8.65	0.002	9.35	0.000
sensitive	TCS	OTU_195	Alphaproteobacteria	4.34	0.002	3.65	0.001
sensitive	TCS	OTU_222	Bacteroidetes	6.33	0.002	6.34	0.000
sensitive	TCS	OTU_90	Actinobacteria	9.46	0.002	9.63	0.000
sensitive	TCS	OTU_180	Gammaproteobacteria	6.21	0.005	4.03	0.019
sensitive	TCS	OTU_359	Alphaproteobacteria	6.84	0.005	7.47	0.000
sensitive	TCS	OTU_65	Actinobacteria	7.92	0.006	8.41	0.000
sensitive	TCS	OTU_162	Bacteroidetes	5.24	0.006	4.52	0.004
sensitive	TCS	OTU_130	Alphaproteobacteria	2.65	0.007	2.13	0.010
sensitive	TCS	OTU_178	Verrucomicrobia	9.03	0.009	9.04	0.001
sensitive	TCS	OTU_136	Planctomycetes	5.47	0.010	9.05	0.000
sensitive	TCS	OTU_412	Alphaproteobacteria	7.18	0.010	7.67	0.001
sensitive	TCS	OTU_164	Gammaproteobacteria	5.18	0.010	7.31	0.000
sensitive	TCS	OTU_135	Alphaproteobacteria	6.66	0.014	6.67	0.003
sensitive	TCS	OTU_245	Bacteroidetes	8.32	0.014	8.32	0.003
sensitive	TCS	OTU_170	Bacteroidetes	5.25	0.016	6.52	0.000
sensitive	TCS	OTU_76	Gammaproteobacteria	2.66	0.016	4.20	0.000
sensitive	TCS	OTU_313	Bacteroidetes	5.18	0.016	7.15	0.000
sensitive	TCS	OTU_160	Actinobacteria	8.98	0.022	8.99	0.005
sensitive	TCS	OTU_235	Planctomycetes	5.64	0.023	6.61	0.001
sensitive	TCS	OTU_395	Bacteroidetes	5.37	0.023	5.35	0.006
sensitive	TCS	OTU_137	Actinobacteria	8.62	0.025	8.63	0.006
sensitive	TCS	OTU_203	Verrucomicrobia	7.31	0.028	7.32	0.007
sensitive	TCS	OTU_262	Alphaproteobacteria	4.76	0.033	4.14	0.021
sensitive	TCS	OTU_218	Gammaproteobacteria	5.10	0.034	6.51	0.001
sensitive	TCS	OTU_190	Bacteroidetes	4.74	0.035	3.84	0.036
sensitive	TCS	OTU_433	Alphaproteobacteria	4.12	0.035	3.68	0.019
sensitive	TCS	OTU_60	Actinobacteria	8.29	0.035	8.30	0.010
sensitive	TCS	OTU_454	Gammaproteobacteria	3.75	0.036	3.39	0.017
sensitive	TCS	OTU_365	Actinobacteria	8.49	0.036	8.50	0.011

Ecological category	Treatment	OTUID	Taxonomy (Phylum/class)	log2(P1/P2)	*P.adj*	log2(P2/P3)	*P.adj*
sensitive	TCS	OTU_278	Verrucomicrobia	6.89	0.036	6.90	0.010
sensitive	TCS	OTU_276	Bacteroidetes	5.49	0.041	7.77	0.001
sensitive	TCS	OTU_16	Planctomycetes	3.19	0.047	4.45	0.001
sensitive	MI	OTU_274	Verrucomicrobia	23.30	0.000	23.62	0.000
sensitive	MI	OTU_19	Planctomycetes	9.26	0.000	8.43	0.000
sensitive	MI	OTU_136	Planctomycetes	8.20	0.000	9.73	0.000
sensitive	MI	OTU_47	Actinobacteria	9.77	0.000	10.88	0.000
sensitive	MI	OTU_27	Actinobacteria	11.48	0.000	11.68	0.000
sensitive	MI	OTU_117	Bacteroidetes	7.62	0.000	4.86	0.001
sensitive	MI	OTU_3	Planctomycetes	5.89	0.000	4.61	0.001
sensitive	MI	OTU_60	Actinobacteria	10.37	0.001	10.09	0.000
sensitive	MI	OTU_104	Actinobacteria	8.47	0.001	8.67	0.000
sensitive	MI	OTU_65	Actinobacteria	10.49	0.002	10.21	0.000
sensitive	MI	OTU_76	Gammaproteobacteria	4.09	0.002	6.00	0.000
sensitive	MI	OTU_72	Gammaproteobacteria	7.17	0.002	8.00	0.000
sensitive	MI	OTU_164	Gammaproteobacteria	3.96	0.002	5.70	0.000
sensitive	MI	OTU_222	Bacteroidetes	6.72	0.002	5.88	0.002
sensitive	MI	OTU_81	Actinobacteria	9.49	0.003	9.21	0.001
sensitive	MI	OTU_332	Bacteroidetes	5.97	0.004	5.04	0.005
sensitive	MI	OTU_366	Bacteroidetes	7.12	0.004	7.32	0.001
sensitive	MI	OTU_131	Alphaproteobacteria	3.87	0.005	4.55	0.000
sensitive	MI	OTU_74	Actinobacteria	9.44	0.005	9.64	0.001
sensitive	MI	OTU_16	Planctomycetes	5.70	0.005	5.47	0.002
sensitive	MI	OTU_235	Planctomycetes	7.94	0.005	8.62	0.000
sensitive	MI	OTU_178	Verrucomicrobia	7.81	0.007	7.53	0.002
sensitive	MI	OTU_113	Planctomycetes	3.49	0.009	9.68	0.000
sensitive	MI	OTU_90	Actinobacteria	8.19	0.009	8.07	0.003
sensitive	MI	OTU_433	Alphaproteobacteria	5.31	0.009	6.11	0.001
sensitive	MI	OTU_467	Bacteroidetes	6.40	0.009	4.22	0.044
sensitive	MI	OTU_170	Bacteroidetes	5.25	0.009	7.54	0.000
sensitive	MI	OTU_308	Actinobacteria	7.75	0.011	7.47	0.005
sensitive	MI	OTU_107	Gammaproteobacteria	2.20	0.015	2.20	0.005
sensitive	MI	OTU_165	Bacteroidetes	7.28	0.015	8.45	0.001
sensitive	MI	OTU_31	Bacteroidetes	4.53	0.015	6.03	0.000
sensitive	MI	OTU_184	Planctomycetes	3.27	0.015	8.79	0.000
sensitive	MI	OTU_200	Planctomycetes	4.49	0.015	8.62	0.000
sensitive	MI	OTU_404	Gammaproteobacteria	4.56	0.017	5.37	0.001
sensitive	MI	OTU_195	Alphaproteobacteria	3.62	0.021	3.88	0.004
sensitive	MI	OTU_9	Alphaproteobacteria	2.12	0.022	1.84	0.023
sensitive	MI	OTU_130	Alphaproteobacteria	2.43	0.022	2.64	0.004
sensitive	MI	OTU_7	Bacteroidetes	3.45	0.023	4.21	0.001

Ecological category	Treatment	OTUID	Taxonomy (Phylum/class)	log2(P1/P2)	*P.adj*	log2(P2/P3)	*P.adj*
sensitive	MI	OTU_75	Bacteroidetes	6.34	0.024	4.84	0.044
sensitive	MI	OTU_239	Bacteroidetes	4.27	0.024	3.53	0.032
sensitive	MI	OTU_135	Alphaproteobacteria	6.44	0.026	7.12	0.004
sensitive	MI	OTU_35	Bacteroidetes	3.48	0.027	3.74	0.006
sensitive	MI	OTU_285	Cyanobacteria	8.28	0.027	8.00	0.014
sensitive	MI	OTU_304	Verrucomicrobia	7.15	0.027	7.03	0.012
sensitive	MI	OTU_244	Actinobacteria	6.89	0.030	6.61	0.016
sensitive	MI	OTU_204	Gammaproteobacteria	3.07	0.031	3.05	0.014
sensitive	MI	OTU_169	Actinobacteria	8.08	0.036	7.80	0.019
sensitive	MI	OTU_137	Actinobacteria	7.94	0.038	8.15	0.013
sensitive	MI	OTU_160	Actinobacteria	7.93	0.038	8.13	0.013
sensitive	MI	OTU_124	Bacteroidetes	3.65	0.045	6.16	0.000
sensitive	MI	OTU_496	Actinobacteria	6.46	0.048	6.18	0.027
sensitive	MII	OTU_16	Planctomycetes	9.52	0.000	10.53	0.000
sensitive	MII	OTU_3	Planctomycetes	8.01	0.000	7.54	0.000
sensitive	MII	OTU_19	Planctomycetes	8.06	0.000	9.42	0.000
sensitive	MII	OTU_131	Alphaproteobacteria	6.62	0.000	4.19	0.002
sensitive	MII	OTU_195	Alphaproteobacteria	7.37	0.000	4.83	0.001
sensitive	MII	OTU_27	Actinobacteria	10.70	0.000	10.60	0.000
sensitive	MII	OTU_47	Actinobacteria	8.81	0.000	10.00	0.000
sensitive	MII	OTU_187	Alphaproteobacteria	6.15	0.000	3.74	0.004
sensitive	MII	OTU_113	Planctomycetes	5.79	0.000	9.58	0.000
sensitive	MII	OTU_104	Actinobacteria	9.14	0.001	8.57	0.000
sensitive	MII	OTU_165	Bacteroidetes	9.51	0.001	8.94	0.000
sensitive	MII	OTU_52	Planctomycetes	6.97	0.001	8.40	0.000
sensitive	MII	OTU_180	Gammaproteobacteria	6.11	0.001	4.04	0.008
sensitive	MII	OTU_72	Gammaproteobacteria	8.76	0.001	9.76	0.000
sensitive	MII	OTU_166	Gammaproteobacteria	7.60	0.002	7.81	0.000
sensitive	MII	OTU_136	Planctomycetes	5.85	0.002	8.73	0.000
sensitive	MII	OTU_154	Verrucomicrobia	4.00	0.002	7.90	0.000
sensitive	MII	OTU_347	Gammaproteobacteria	8.28	0.003	7.86	0.001
sensitive	MII	OTU_74	Actinobacteria	9.35	0.005	8.77	0.002
sensitive	MII	OTU_262	Alphaproteobacteria	5.42	0.006	4.23	0.011
sensitive	MII	OTU_76	Gammaproteobacteria	4.77	0.006	3.81	0.011
sensitive	MII	OTU_90	Actinobacteria	9.37	0.008	9.28	0.002
sensitive	MII	OTU_257	Planctomycetes	5.76	0.009	7.19	0.000
sensitive	MII	OTU_65	Actinobacteria	8.08	0.009	7.98	0.002
sensitive	MII	OTU_222	Bacteroidetes	7.84	0.010	7.26	0.004
sensitive	MII	OTU_460	Verrucomicrobia	6.77	0.011	6.19	0.005
sensitive	MII	OTU_164	Gammaproteobacteria	5.28	0.011	7.20	0.000
sensitive	MII	OTU_81	Actinobacteria	8.50	0.012	8.40	0.003

Ecological category	Treatment	OTUID	Taxonomy (Phylum/class)	log2(P1/P2)	*P.adj*	log2(P2/P3)	*P.adj*
sensitive	MII	OTU_448	Planctomycetes	6.56	0.012	5.98	0.007
sensitive	MII	OTU_137	Actinobacteria	7.23	0.013	7.14	0.004
sensitive	MII	OTU_197	Alphaproteobacteria	8.42	0.013	8.32	0.004
sensitive	MII	OTU_357	Bacteroidetes	5.37	0.016	5.45	0.003
sensitive	MII	OTU_235	Planctomycetes	6.12	0.016	7.99	0.000
sensitive	MII	OTU_135	Alphaproteobacteria	9.22	0.017	8.65	0.008
sensitive	MII	OTU_470	Gammaproteobacteria	4.41	0.020	4.91	0.002
sensitive	MII	OTU_60	Actinobacteria	8.90	0.027	8.33	0.015
sensitive	MII	OTU_358	Bacteroidetes	7.19	0.030	6.62	0.018
sensitive	MII	OTU_379	Actinobacteria	6.82	0.039	6.25	0.023
sensitive	MII	OTU_178	Verrucomicrobia	7.23	0.041	7.63	0.008
sensitive	MII	OTU_160	Actinobacteria	7.82	0.041	7.25	0.023
sensitive	MII	OTU_224	Actinobacteria	8.14	0.043	7.57	0.024
tolerant	BPA	OTU_109	Alphaproteobacteria	-6.09	0.000	-3.38	0.002
tolerant	BPA	OTU_48	Alphaproteobacteria	-10.09	0.000	-11.95	0.000
tolerant	BPA	OTU_78	Gammaproteobacteria	-6.09	0.000	-8.93	0.000
tolerant	BPA	OTU_14	Verrucomicrobia	-7.57	0.000	-6.63	0.003
tolerant	BPA	OTU_186	Bacteroidetes	-6.82	0.005	-5.89	0.047
tolerant	BPA	OTU_134	Bacteroidetes	-3.59	0.010	-4.81	0.003
tolerant	TCS	OTU_48	Alphaproteobacteria	-10.34	0.000	-10.30	0.000
tolerant	TCS	OTU_78	Gammaproteobacteria	-7.98	0.000	-8.42	0.000
tolerant	TCS	OTU_215	Bacteroidetes	-9.44	0.000	-4.81	0.022
tolerant	TCS	OTU_273	Bacteroidetes	-8.76	0.000	-6.79	0.004
tolerant	TCS	OTU_250	Verrucomicrobia	-5.90	0.000	-3.85	0.047
tolerant	TCS	OTU_306	Gammaproteobacteria	-7.14	0.000	-7.11	0.004
tolerant	TCS	OTU_372	Gammaproteobacteria	-7.10	0.001	-6.10	0.013
tolerant	TCS	OTU_75	Bacteroidetes	-4.05	0.002	-4.91	0.002
tolerant	TCS	OTU_102	Bacteroidetes	-3.94	0.002	-6.17	0.000
tolerant	TCS	OTU_307	Planctomycetes	-3.09	0.004	-2.69	0.037
tolerant	TCS	OTU_33	Alphaproteobacteria	-3.30	0.004	-3.77	0.005
tolerant	TCS	OTU_138	Gammaproteobacteria	-3.27	0.015	-3.31	0.047
tolerant	TCS	OTU_134	Bacteroidetes	-3.05	0.015	-3.43	0.022
tolerant	TCS	OTU_216	Gammaproteobacteria	-4.57	0.028	-5.87	0.022
tolerant	TCS	OTU_279	Gammaproteobacteria	-6.72	0.029	-22.59	0.000
tolerant	MI	OTU_78	Gammaproteobacteria	-9.44	0.000	-8.99	0.000
tolerant	MI	OTU_48	Alphaproteobacteria	-11.47	0.000	-11.48	0.000
tolerant	MI	OTU_33	Alphaproteobacteria	-4.84	0.000	-3.60	0.026
tolerant	MI	OTU_116	Chlamydiae	-8.16	0.001	-7.39	0.005
tolerant	MI	OTU_252	Bacteroidetes	-7.95	0.001	-5.38	0.047
tolerant	MI	OTU_128	Gammaproteobacteria	-7.43	0.002	-7.98	0.004
tolerant	MI	OTU_156	Bacteroidetes	-4.16	0.010	-4.01	0.031

Ecological category	Treatment	OTUID	Taxonomy (Phylum/class)	log2(P1/P2)	*P.adj*	log2(P2/P3)	*P.adj*
tolerant	MI	OTU_146	Verrucomicrobia	-6.91	0.014	-7.40	0.025
tolerant	MI	OTU_253	Bacteroidetes	-5.74	0.017	-6.17	0.027
tolerant	MI	OTU_49	Bacteroidetes	-5.05	0.017	-5.25	0.031
tolerant	MI	OTU_306	Gammaproteobacteria	-5.32	0.020	-5.81	0.029
tolerant	MI	OTU_490	Verrucomicrobia	-5.62	0.021	-6.11	0.031
tolerant	MII	OTU_211	Bacteroidetes	-22.86	0.000	-7.68	0.010
tolerant	MII	OTU_44	Alphaproteobacteria	-24.61	0.000	-9.16	0.014
tolerant	MII	OTU_151	Alphaproteobacteria	-23.63	0.000	-27.79	0.000
tolerant	MII	OTU_78	Gammaproteobacteria	-8.44	0.000	-8.25	0.000
tolerant	MII	OTU_58	Gammaproteobacteria	-6.53	0.000	-3.45	0.024
tolerant	MII	OTU_48	Alphaproteobacteria	-10.14	0.000	-10.51	0.000
tolerant	MII	OTU_134	Bacteroidetes	-7.74	0.000	-5.92	0.000
tolerant	MII	OTU_306	Gammaproteobacteria	-6.50	0.000	-6.40	0.000
tolerant	MII	OTU_77	Alphaproteobacteria	-9.15	0.001	-8.55	0.010
tolerant	MII	OTU_372	Gammaproteobacteria	-6.10	0.009	-6.48	0.022

Table S4 The list of ecologically-grouped OTUs (opportunistic, sensitive and tolerant) shared across treatments. Max_rel: maximal relative abundance of the OTUs in all samples; max_id: the sample ID in which the maximal relative abundance was detected, e.g., denoted by "batch_treatment_replicate", sample 'B1_CON_002' represents the number of batch, treatment id and the number of the biological replicate in that treatment. Treatment IDs are: 'Initial' containing the starting bacterial communities before the experimental implementation; 'Con' microcosms containing no micropollutant additions and serve as the controls; 'BPA' microcosms containing bisphenol A; 'TCS' microcosms containing triclosan; 'MI' microcosms containing a mixture of bisphenol A and triclosan; 'MII' microcosms containing a mixture of bisphenol A, triclosan, bisphenol-S and triclocarban.

OTUID	Treatment				Taxonomic affiliation					max_rel	max_id
	BPA	TCS	MI	MII	Phylum	Class	Order	Family	Genus		
OTU_104	sensitive	sensitive	sensitive	sensitive	Actinobacteria	Actinobacteria	Frankiales	Sporichthyaceae	hgcI clade	6.34%	Initial_002
OTU_137	sensitive	sensitive	sensitive	sensitive	Actinobacteria	Actinobacteria	Frankiales	Sporichthyaceae	Ambiguous_taxa	1.66%	Initial_002
OTU_158	sensitive	sensitive			Actinobacteria	Actinobacteria	Frankiales	Sporichthyaceae	Candidatus Planktophila	4.29%	Initial_002
OTU_160	sensitive	sensitive	sensitive	sensitive	Actinobacteria	Actinobacteria	Frankiales	Sporichthyaceae	hgcI clade	0.97%	Initial_001
OTU_169	sensitive		sensitive		Actinobacteria	Actinobacteria	Frankiales	Sporichthyaceae	hgcI clade	3.86%	B1_BPA_001
OTU_27	sensitive	sensitive	sensitive	sensitive	Actinobacteria	Actinobacteria	Frankiales	Sporichthyaceae	hgcI clade	15.94%	Initial_002
OTU_308	sensitive		sensitive		Actinobacteria	Actinobacteria	Corynebacteriales	Mycobacteriaceae	Mycobacterium	0.28%	B1_BPA_001
OTU_379	sensitive			sensitive	Actinobacteria	Thermoleophilia	Solirubrobacterales	Solirubrobacteraceae	Conexibacter	0.16%	B1_MII_001
OTU_47	sensitive	sensitive	sensitive	sensitive	Actinobacteria	Actinobacteria	Frankiales	Sporichthyaceae	hgcI clade	8.32%	Initial_002
OTU_60	sensitive	sensitive	sensitive	sensitive	Actinobacteria	Acidimicrobiia	Microtrichales	Ilumatobacteraceae	CL500-29 marine group	7.72%	Initial_003
OTU_65	sensitive	sensitive	sensitive	sensitive	Actinobacteria	Actinobacteria	Frankiales	Sporichthyaceae	hgcI clade	11.65%	Initial_001
OTU_74	sensitive	sensitive	sensitive	sensitive	Actinobacteria	Actinobacteria	Frankiales	Sporichthyaceae	hgcI clade	13.28%	Initial_002
OTU_81	sensitive	sensitive	sensitive	sensitive	Actinobacteria	Actinobacteria	Frankiales	Sporichthyaceae		4.46%	Initial_002
OTU_90	sensitive	sensitive	sensitive	sensitive	Actinobacteria	Acidimicrobiia	Microtrichales	Ilumatobacteraceae	CL500-29 marine group	5.01%	Initial_002
OTU_130		sensitive	sensitive		Proteobacteria	Alphaproteobacteria	Rhizobiales	Beijerinckiaceae	Methylobacterium	0.56%	Initial_002
OTU_131	sensitive	sensitive	sensitive	sensitive	Proteobacteria	Alphaproteobacteria	Rhizobiales	Beijerinckiaceae	Methylobacterium	0.86%	B1_MII_003
OTU_135	sensitive	sensitive	sensitive	sensitive	Proteobacteria	Alphaproteobacteria	SAR11 clade	Clade III	uncultured bacterium	2.60%	Initial_001
OTU_187	sensitive	sensitive		sensitive	Proteobacteria	Alphaproteobacteria	Caulobacterales	Caulobacteraceae	Caulobacter	0.40%	B1_MII_003
OTU_195	sensitive	sensitive	sensitive	sensitive	Proteobacteria	Alphaproteobacteria	Rhizobiales	Beijerinckiaceae	Methylobacterium	0.76%	B1_MII_003
OTU_33		tolerant	tolerant		Proteobacteria	Alphaproteobacteria	Sphingomonadales	Sphingomonadaceae	Novosphingobium	30.48%	B6_BPA_001
OTU_433		sensitive	sensitive		Proteobacteria	Alphaproteobacteria	Rhizobiales	Beijerinckiaceae	Methylobacterium	0.14%	B1_MI_003
OTU_48	tolerant	tolerant	tolerant	tolerant	Proteobacteria	Alphaproteobacteria	Sphingomonadales	Sphingomonadaceae	Sphingomonas	9.71%	B7_MI_001

OTUID	Treatment				Taxonomic affiliation					max_rel	max_id
	BPA	TCS	MI	MII	Phylum	Class	Order	Family	Genus		
OTU_9	sensitive		sensitive		Proteobacteria	Alphaproteobacteria	Rhizobiales	Rhizobiaceae	Shinella	9.26%	B1_MII_002
OTU_107	sensitive		sensitive		Proteobacteria	Gammaproteobacteria	Betaproteobacteriales	Rhodocyclaceae	Methyloversatilis	0.66%	B4_TCS_001
OTU_164	sensitive	sensitive	sensitive	sensitive	Proteobacteria	Gammaproteobacteria	Betaproteobacteriales	Burkholderiaceae	MWH-UniP1 aquatic group	0.83%	B1_MII_002
OTU_166	sensitive			sensitive	Proteobacteria	Gammaproteobacteria	Betaproteobacteriales	Burkholderiaceae	Polynucleobacter	3.42%	B1_MII_001
OTU_180		sensitive		sensitive	Proteobacteria	Gammaproteobacteria	Betaproteobacteriales	Methylophilaceae	Methylotenera	0.43%	B1_CON_002
OTU_470	sensitive			sensitive	Proteobacteria	Gammaproteobacteria	Betaproteobacteriales	Nitrosomonadaceae	Ellin6067	0.04%	B1_BPA_001
OTU_72	sensitive	sensitive	sensitive	sensitive	Proteobacteria	Gammaproteobacteria	Betaproteobacteriales	Burkholderiaceae		2.71%	B1_BPA_002
OTU_76	sensitive	sensitive	sensitive	sensitive	Proteobacteria	Gammaproteobacteria	Betaproteobacteriales	Burkholderiaceae	Polynucleobacter	4.13%	B1_MII_001
OTU_78	tolerant	tolerant	tolerant	tolerant	Proteobacteria	Gammaproteobacteria	Betaproteobacteriales	Burkholderiaceae	Hydrogenophaga	4.26%	B6_CON_003
OTU_306		tolerant	tolerant	tolerant	Proteobacteria	Gammaproteobacteria	Xanthomonadales	Xanthomonadaceae	Silanimonas	0.14%	B5_MI_003
OTU_372		tolerant		tolerant	Proteobacteria	Gammaproteobacteria	Xanthomonadales	Xanthomonadaceae	Silanimonas	0.17%	B5_TCS_003
OTU_117	sensitive	sensitive	sensitive		Bacteroidetes	Bacteroidia	Flavobacteriales	Flavobacteriaceae	Flavobacterium	2.66%	B1_CON_001
OTU_134	tolerant	tolerant		tolerant	Bacteroidetes	Bacteroidia	Flavobacteriales	Flavobacteriaceae	Flavobacterium	1.24%	B6_MII_001
OTU_165	sensitive	sensitive	sensitive	sensitive	Bacteroidetes	Bacteroidia	Sphingobacteriales	env.OPS 17		1.01%	B1_BPA_002
OTU_170	sensitive	sensitive	sensitive		Bacteroidetes	Bacteroidia	Chitinophagales	Chitinophagaceae		1.43%	B6_MI_002
OTU_185	sensitive	sensitive			Bacteroidetes	Bacteroidia	Sphingobacteriales			0.33%	B2_BPA_001
OTU_222	sensitive	sensitive	sensitive	sensitive	Bacteroidetes	Bacteroidia	Chitinophagales	37-13		1.18%	B2_BPA_001
OTU_245	sensitive	sensitive			Bacteroidetes	Bacteroidia	Sphingobacteriales	env.OPS 17		1.07%	B1_BPA_002
OTU_313	sensitive	sensitive			Bacteroidetes	Bacteroidia	Chitinophagales	Chitinophagaceae		0.34%	B1_MI_002
OTU_332		sensitive	sensitive		Bacteroidetes	Bacteroidia	Sphingobacteriales			0.17%	B1_TCS_001
OTU_357	sensitive			sensitive	Bacteroidetes	Bacteroidia	Flavobacteriales	Flavobacteriaceae	Flavobacterium	0.31%	B1_MI_002
OTU_366	sensitive		sensitive		Bacteroidetes	Bacteroidia	Chitinophagales	Chitinophagaceae	Heliimonas	0.35%	B1_BPA_003
OTU_75		tolerant	sensitive		Bacteroidetes	Bacteroidia	Chitinophagales	Chitinophagaceae		11.06%	B6_TCS_002

OTUID	Treatment				Taxonomic affiliation					max_rel	max_id
	BPA	TCS	MI	MII	Phylum	Class	Order	Family	Genus		
OTU_285	sensitive		sensitive		Cyanobacteria	Oxyphotobacteria	Synechococcales	Cyanobiaceae	Cyanobium PCC-6307	0.38%	Initial_001
OTU_113	sensitive		sensitive	sensitive	Planctomycetes	Planctomycetacia	Planctomycetales	Schlesneriaceae	Planctopirus	1.41%	B1_MII_003
OTU_136	sensitive	sensitive	sensitive	sensitive	Planctomycetes	Planctomycetacia	Pirellulales	Pirellulaceae	uncultured	0.95%	B1_MII_001
OTU_16		sensitive	sensitive	sensitive	Planctomycetes	Planctomycetacia	Pirellulales	Pirellulaceae	Pirellula	16.49%	B1_MII_003
OTU_19	sensitive		sensitive	sensitive	Planctomycetes	Planctomycetacia	Pirellulales	Pirellulaceae	Pirellula	9.59%	B2_MI_002
OTU_235		sensitive	sensitive	sensitive	Planctomycetes	Planctomycetacia	Pirellulales	Pirellulaceae	uncultured	0.49%	B1_CON_003
OTU_3	sensitive		sensitive	sensitive	Planctomycetes	Planctomycetacia	Pirellulales	Pirellulaceae	Pirellula	27.65%	B2_CON_003
OTU_52	sensitive			sensitive	Planctomycetes	Planctomycetacia	Pirellulales	Pirellulaceae	Pirellula	5.42%	B3_TCS_001
OTU_154	sensitive			sensitive	Verrucomicrobia	Verrucomicrobiae	Verrucomicrobiales	Verrucomicrobiaceae	uncultured	0.56%	B2_CON_003
OTU_178	sensitive	sensitive	sensitive	sensitive	Verrucomicrobia	Verrucomicrobiae	Methylacidiphilales	Methylacidiphilaceae	uncultured	0.95%	B1_TCS_002
OTU_203	sensitive	sensitive			Verrucomicrobia	Verrucomicrobiae	Methylacidiphilales	Methylacidiphilaceae	uncultured	1.17%	Initial_002
OTU_274	sensitive		sensitive		Verrucomicrobia	Verrucomicrobiae	Methylacidiphilales	Methylacidiphilaceae	uncultured	0.56%	B1_MI_002
OTU_278	sensitive	sensitive			Verrucomicrobia	Verrucomicrobiae	Methylacidiphilales	Methylacidiphilaceae	uncultured	0.57%	Initial_001

Figure S1. The location of the Shidou Reservoir in the map of Xiamen city (A) and sampling site in the reservoir (B).

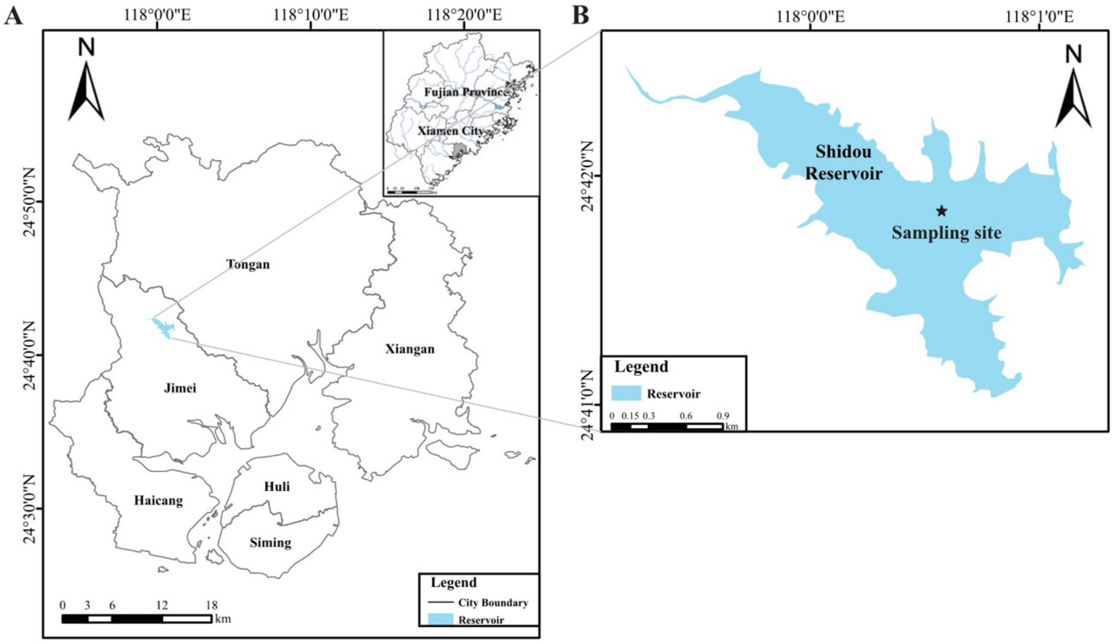

Figure S2 The removal rates of the micropollutants in sterile microcosms spiked with1 μg/L bisphenol A (BPA), 0.1 μg/L bisphenol S (BPS), 1 μg/L triclosan (TCS) and 0.1 μg/L triclocarban (TCC) for 7-day incubation. The removal rates were presented as the average of the triplicates.

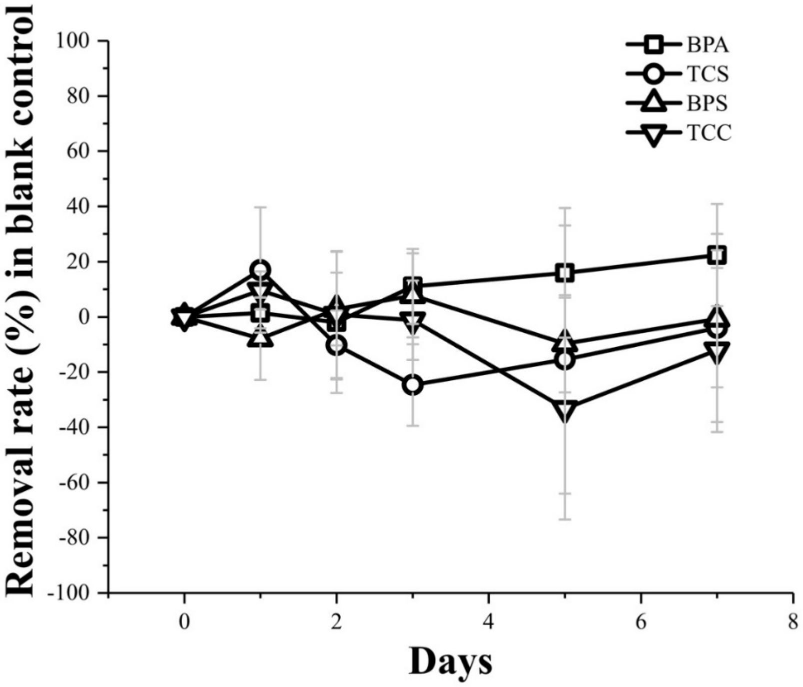

Figure S3 The variations in concentration of 1 mg/L bisphenol A (BPA), 1 mg/L triclosan (TCS) and 1 mg/L mixed BPA+TCS under the bio-transformation of prokaryotic cultures before (a) and after (b) microcosm cultivation at 1 μg/L level. The prokaryotic cultures used in 1 mg/L bio-transformation experiments were transferred from 1 μg/L microcosm group BPA, TCS and MI respectively. Curves in black and red represent the concentration variations of BPA and TCS in the respective treatment, respectively.

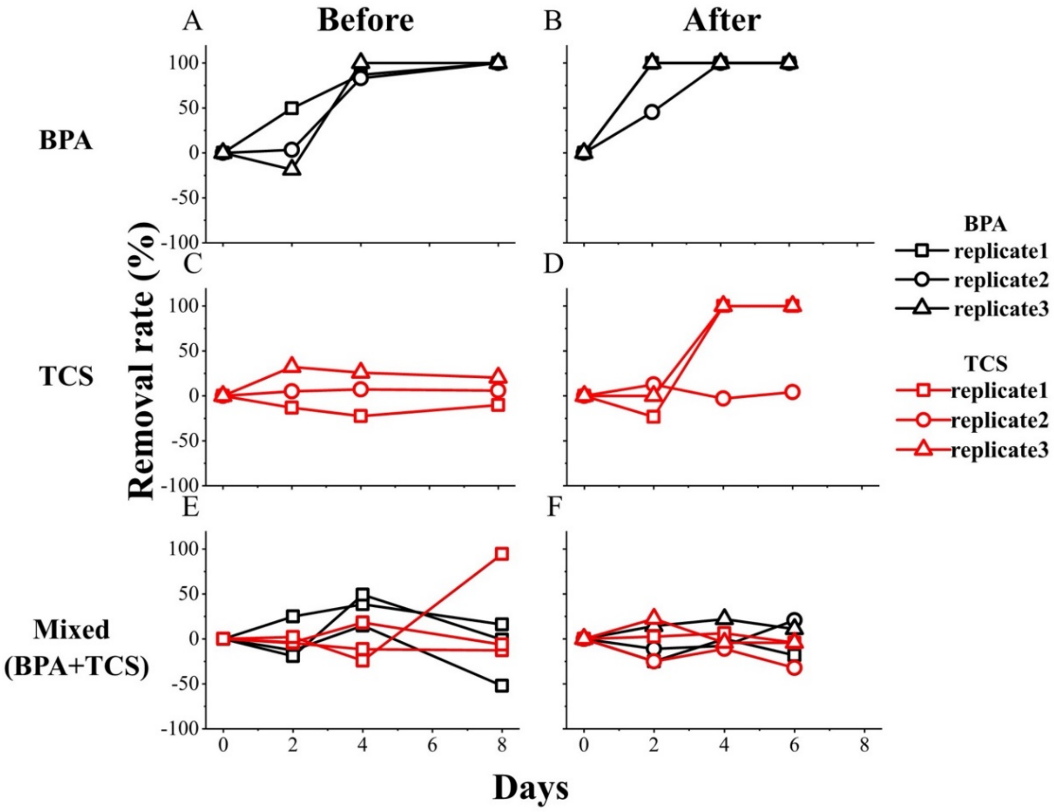

Figure S4. The temporal variation of abundance-weighted average 16S rRNA gene copy numbers of bacterial communities in the controls and micropollutant-treated microcosms (A-E), of Pearson's correlation for pairwise treatment comparison (F) and the grouping of similar frequency of gene copy numbers (G). Color gradients in (F) represent Pearson's correlation coefficients. A hierarchical dendrogram (G) illustrated the similar frequency (based on Bray-Curtis dissimilarity) of 16S rRNA gene copy numbers of the communities in the micropollutants-treated batches. Three growth progression phrases across all microcosm batches were identified based on similar average gene copy numbers and the time continuity of the inoculation phase 1 (B1–B2), phase 2 (B3–B4), and phase 3 (B5–B7).

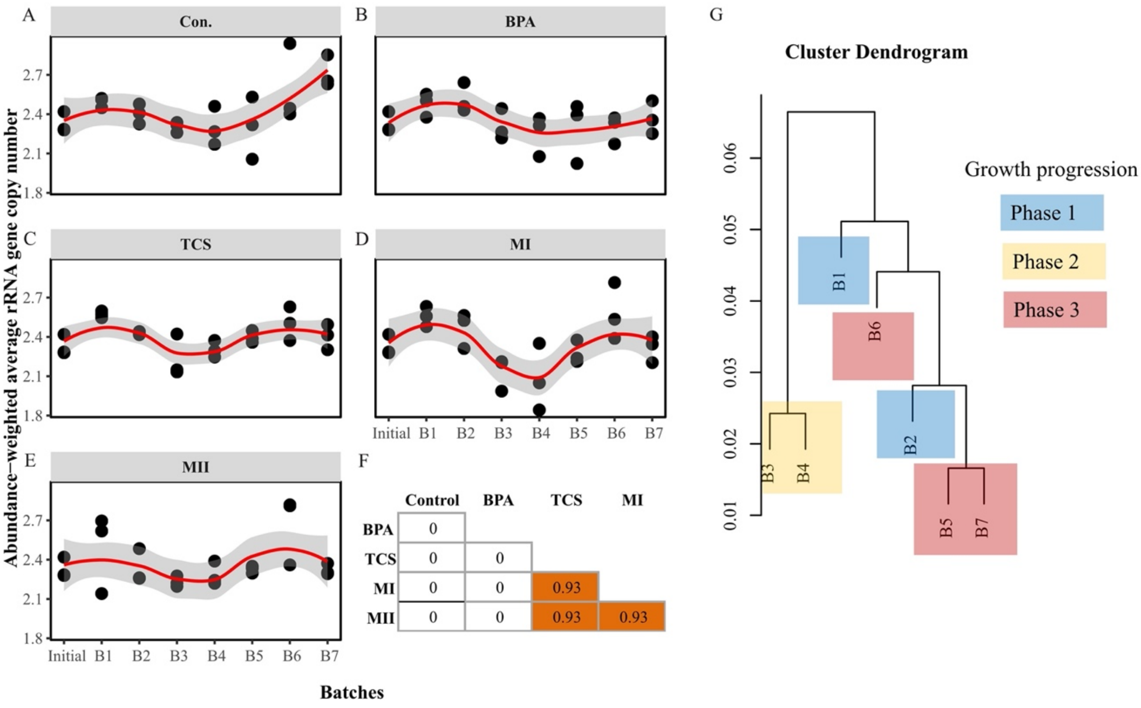

Figure S5 Percent removal of bisphenol A (BPA), triclosan (TCS), bisphenol S (BPS), and triclocarbon (TCC) from the microcosms. (A) Removal in the single-micropollutant (BPA and TCA) treatments; (B) removal in the mixed-micropollutant (MI and MII) treatments. The open circles represent the removal percentage obtained from each microcosm batch. Tukey's *post-hoc* test was used to determine at which micropollutant the differences in rates occurred, and in this case the removal rate of the micropollutants from each microcosm batch was used as replicates for the test. The corresponding homogeneous groups were determined at significance level $P < 0.05$ and are indicated by a and b.

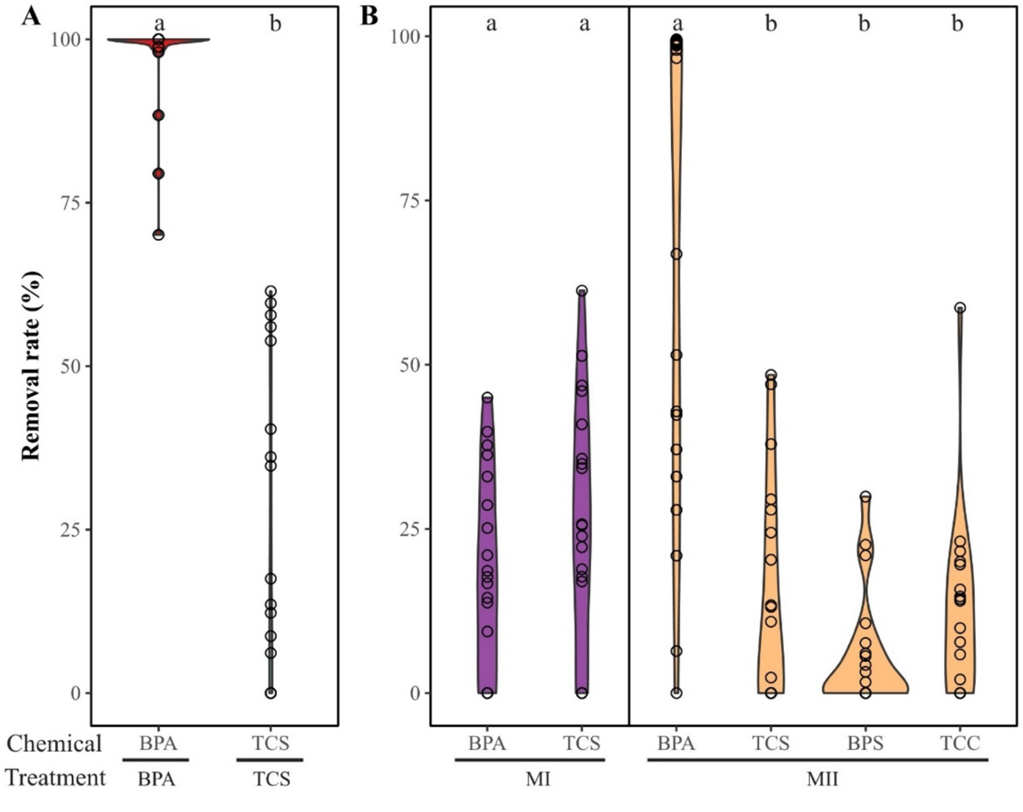

Figure S6. Non-metric multidimensional scaling (NMDS) based on Bray-Curtis dissimilarity matric illustrating the beta-diversity (between-sample differences in community composition) of the initial inoculum, the controls, and the treatments. The analysis was done using the exact sequence variants generated from DADA2 pipeline (Callahan et al., 2017). Raw sequences were processed using the DADA2 pipeline according to the DADA2 tutorial (v1.14) in R. The primers of sequences were trimmed using Cutadapt, and the resulting sequences were quality filtered with customized modifications as follows: truncLen=c(220, 190), maxEE=2, truncQ=2, maxN=0, rm.phix=TRUE, trimLeft=c(0,0). Subsequently, denoising, merging and chimera removal were completed according to the DADA2 pipeline tutorial. All sequences were aligned and assigned taxonomically using the SILVA v.132 reference database. The M^2 value and *P*-value were obtained from Procrustes statistic: M^2 value represents sum of squared deviations between sample pairs, and the lower value mean a better fit between matrices.

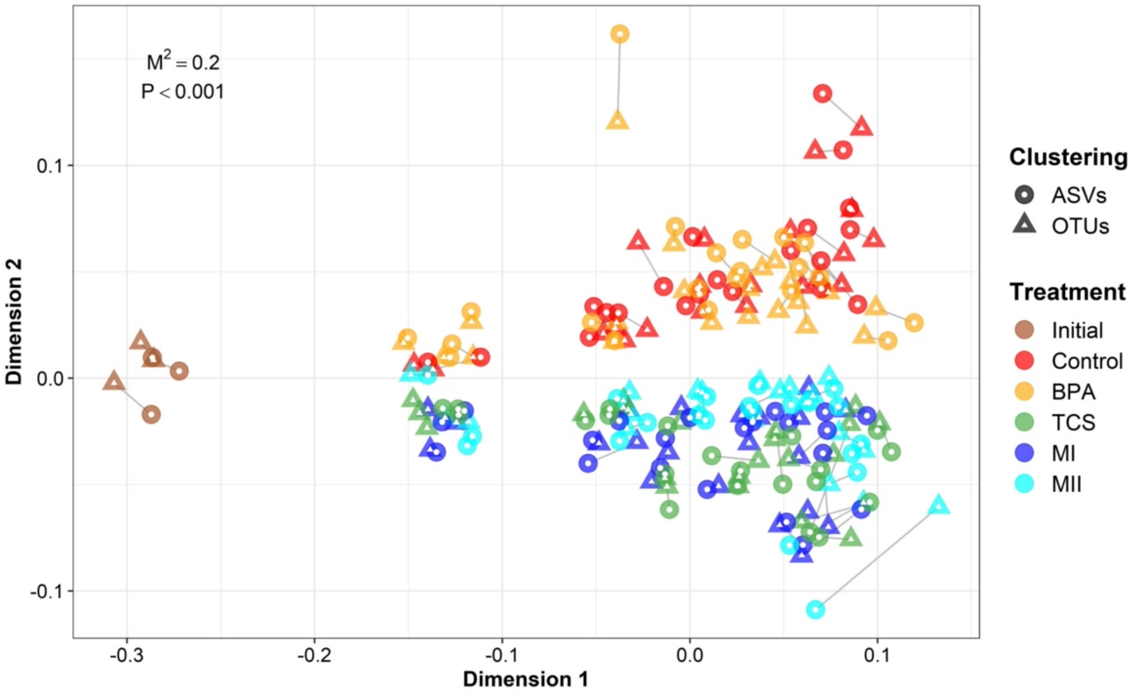

Reference:

Callahan BJ, McMurdie PJ, and Holmes SP. (2017). Exact sequence variants should replace operational taxonomic units in marker-gene data analysis. *ISME J*, 11:2639–2643.

Figure S7. Bacterial community dissimilarity between the controls and micropollutant-treated microcosms (A-D) and the degree of variances between slopes indicated by |t| values (E). In A-D panels, the slopes (*v*) indicate rates of divergent succession of the micropollutant-treated communities from the control communities, estimated with a linear regression fitting between treatment-versus-control distance and time. In E panel, the significance of each linear regression is tested by permutation (1000 iterations), and significance level was determined at $P < 0.05$. The significant variances between slopes are estimated by bootstrapping (1000 iterations), followed by pairwise t-test with Bonferroni correction ($P < 0.05$).

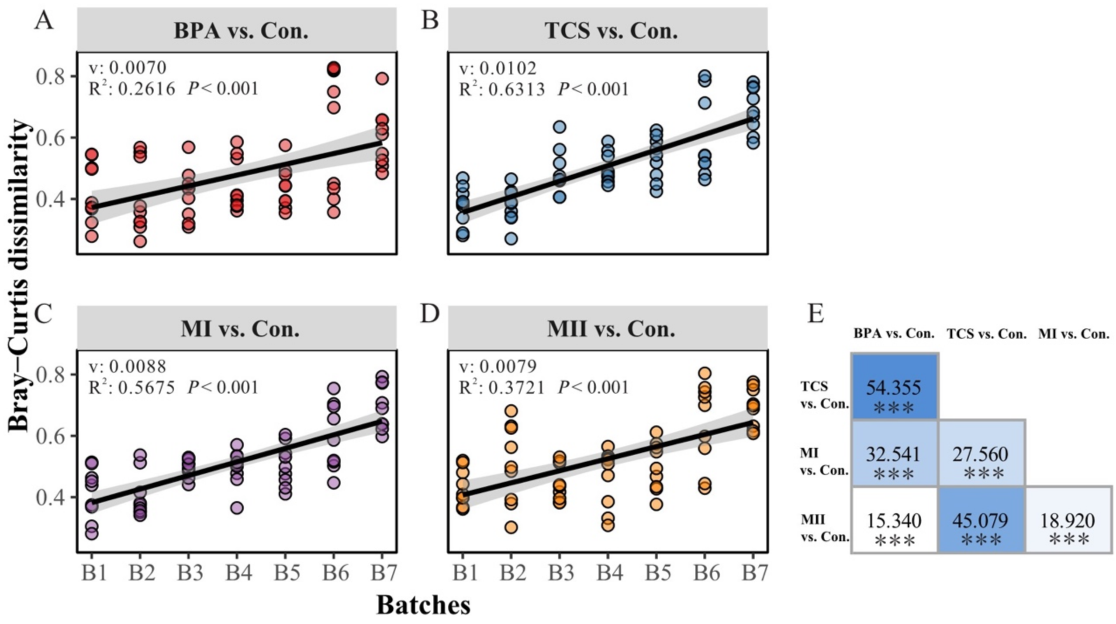

Figure S8. Relative abundance of the sensitive and tolerant taxa in each microcosm batch. Within each ecological group, the relative abundance was presented by the sum of the OTUs assigned to that group. Three growth progression phrases across all microcosm batches were identified based on similar average gene copy numbers and the time continuity of the inoculation phase 1 (B1–B2), phase 2 (B3–B4), and phase 3 (B5–B7).

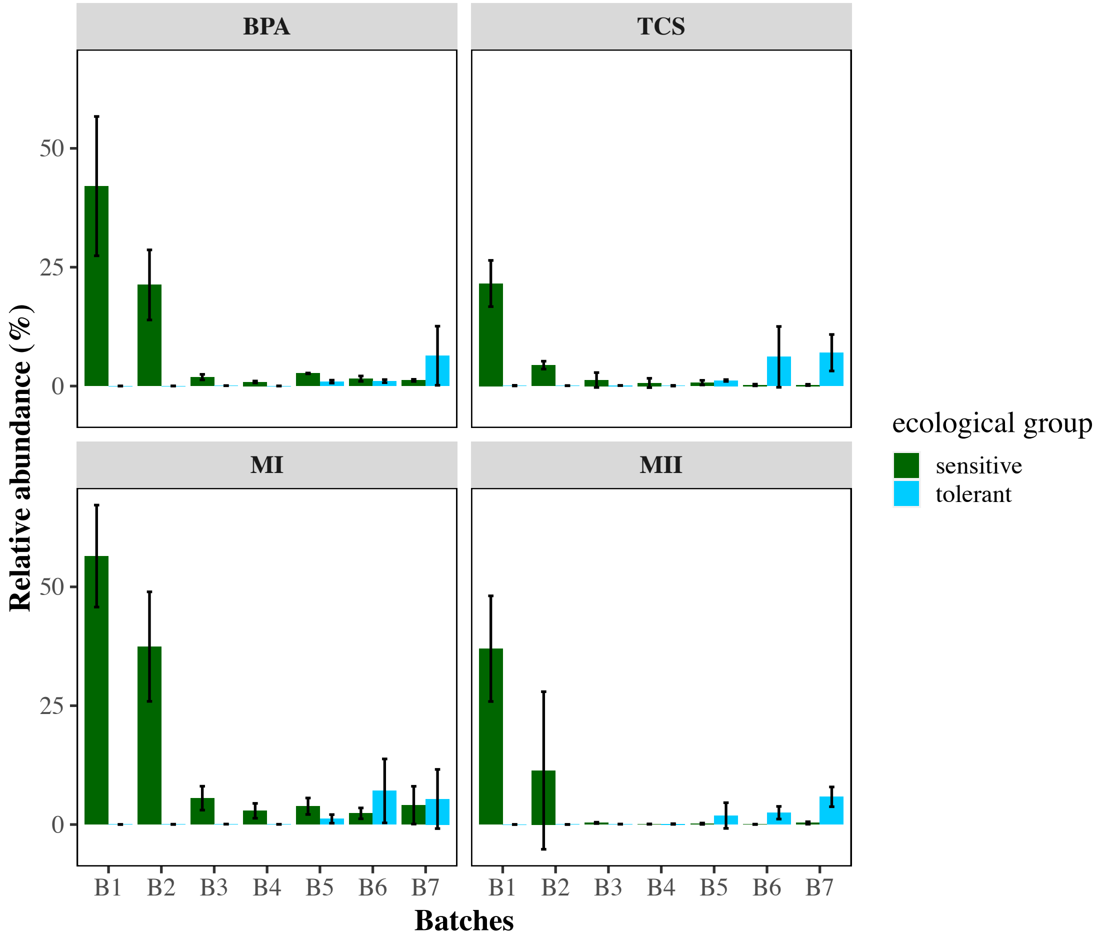

Figure S9 Venn diagram illustrating the unique fraction of bacterial OTUs assigned to ecological categories found in the treatments but not shared in the controls when analyzed with the same filtering criteria as done for ecological grouping. The percentage in each sector of the Venn diagram presents the proportion of OTUs that are unique to either the controls or treatments, or that are shared between the two. A large fraction of the bacterial OTUs was unique to each of three ecological categories. This suggests that the identification of ecological strategies was not biased towards the selection of bacterial taxa whose high abundance in each defined phase was due to simple enrichment in the controls over time.

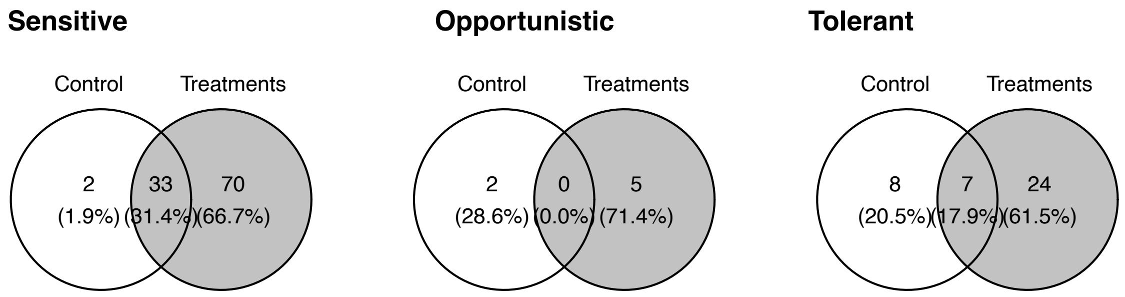

Supplement: Supplementary file 1 — Supplementary information [file 43705_2022_129_MOESM1_ESM.pdf]
